# Impact of cervical cancer–related stigma on screening uptake in Dakar and Kedougou, Senegal: A context-specific stigma index

**DOI:** 10.1371/journal.pgph.0006187

**Published:** 2026-10-08

**Authors:** Fatou Bintou Seck, Saria Awadalla, Fatoumata Binetou Diongue, Ibrahima Ndiaye, Omar Gassama, Ndeye Mbombe Dieng, Adja Maguette Diaw, Hilaire Sarr, Victoria Winslow, Ndèye Marème Sougou, Sarah Abboud, Assane Sall, Alexis Claire Klima, Aida Fall, Aida Barro, Blessings Chisunkha, Ahmadou Babana El Alaoui, Aliath Salami, Jon Andrew Dykens, Caryn Elizabeth Peterson, Adama Faye

**Affiliations:** 1 Institute of Health and Development, Cheikh Anta Diop University, Dakar, Senegal; 2 Division of Epidemiology & Biostatistics, School of Public Health, University of Illinois Chicago, Chicago, Illinois, United States of America; 3 Obstetrics and Gynecology, Aristide Le Dantec Teaching Hospitals, Cheikh Anta Diop University, Dakar, Senegal; 4 Center for Training in Women’s Health, Cheikh Anta Diop University, Dakar, Senegal; 5 Office of Cancer Prevention and Control, Division of Non-Communicable Diseases, Ministry of Health and Social Action, Dakar, Senegal; 6 Office of Planning, Monitoring, Evaluation, and Research, Division of Maternal and Child Health, Ministry of Health and Social Action, Dakar, Senegal; 7 School of Public Health, Epidemiology Division, University of Illinois Chicago, Chicago, Illinois, United States of America; 8 Department of Human Development Nursing Science, College of Nursing, University of Illinois Chicago, Chicago, Illinois, United States of America; 9 College of Applied Health Sciences, University of Illinois Chicago, Chicago, Illinois, United States of America; 10 Department of Biomedical & Health Information Sciences, College of Applied Health Sciences, University of Illinois Chicago, Chicago, Illinois, United States of America; 11 College of Medicine, University of Illinois Chicago, Chicago, Illinois, United States of America; 12 Department of Family and Community Medicine, University of Illinois Chicago, Chicago, Illinois, United States of America; 13 Center for Global Health at the College of Medicine, University of Illinois Chicago, Chicago, Illinois, United States of America; 14 Cancer Center, University of Illinois Chicago, Chicago, Illinois, United States of America; PLOS: Public Library of Science, UNITED STATES OF AMERICA

## Abstract

Cervical cancer is a major public health problem, but it can be prevented through screening and treatment of precancerous lesions. The aim of this study was to assess the association between cervical cancer-related stigma and prior cervical cancer screening uptake among women in the Dakar and Kédougou regions of Senegal. A quantitative descriptive and analytical cross-sectional study was carried out in the Dakar and Kédougou regions. The study population consisted of women aged 25–69. Sampling was based on a two-stage cluster sampling methodology. Past participation in screening was self-reported, and an index of stigma was developed to quantify the level of cervical cancer related stigma in the study population. The items used to develop this index covered several domains that have been shown to be powerful predictors of stigma. Data were analyzed with R 4.5.0 software. Logistic regression was performed (5% alpha). A total of 287 women had data on their screening status, with only 15.7% reporting having undergone one or more screenings in the past. The mean age of participants was 36.23, and the majority (68.6%) were married and living in monogamous households. After adjustment for age, marital status and education level, higher stigma scores were associated with lower odds of screening (OR=0.58, 95% CI 0.38-0.83, p = 0.006). Cervical cancer stigma was significantly associated with lower odds of screening uptake. A better understanding of this barrier will enable us to better adapt strategies to combat this disease. Setting up a navigation program for this screening could help improve access to this service by removing the stigma barrier and any other major obstacle to screening. Our stigma index provides a comprehensive assessment of stigma and represents a novel tool that can be adapted for future research, including the evaluation of stigma reduction following implementation of our patient navigation intervention.

## Introduction

Worldwide, cervical cancer is the fourth most common cancer in women, with an estimated 661,021 new cases and 348,189 deaths per year in 2022 [1]. Cervical cancer is a significant public health problem, especially in low- and middle-income countries (LMICs) [2]. Cervical cancer incidence is rising steadily in sub-Saharan Africa, where there are over 75,000 new cases and nearly 50,000 deaths per year [3], and more than 80% of cervical cancer deaths occur in LMICs due to the existing inequities in wealth, gender, and access to health services [4]. Senegal has the 17th highest cervical cancer incidence, globally [5]. The prevalence of this cancer in Senegal in 2023 was 12.6% where it is the leading cause of cancer-related death among women [1]. Cervical cancer is a highly preventable disease which, in LMICs, is typically diagnosed at a late stage [6]. Vaccination against Human Papillomavirus (HPV) and the early detection of precancerous lesions are by far the most effective strategies for combating cervical cancer [5]. In Senegal, the screening rate remains very low. In 2020, the estimated participation rate in cervical cancer screening was 6.9% among women aged 18–69. Screening is especially low in rural regions of the country and in older age groups (1.9% of women aged 40–49 and 0% for women aged 50 and over) [7].

Stigma is a well-documented barrier to care-seeking behavior, engagement in care, and adherence to treatment in a range of health problems worldwide [8]. It may be influenced by income, ethnicity, gender norms, culture, and type of cancer, and women suffer from additional stigma when cancers are linked to their reproductive system and in contexts where traditional gender roles exist [9,10]. Early research on health-related stigma has primarily focused on patient-centered experiences of stigma and emphasized its conceptual multidimensionality, with stigma posited to be composed of six components: peril or perceived danger from the stigmatized person; the course or temporal changes of the disease; the origin or source of disease; concealability of the disease; the degree it disrupts interactions with others; and its aesthetics or how it might be visually perceived by others [11]. More recent studies have identified two key issues surrounding stigma research, namely the dearth of studies focusing on perceptions of stigma from the non-afflicted general public as opposed to patient-centered stigma [12] and, pertinent to this study, the development of a validated scale to quantify perceptions of stigma in non-afflicted populations [13].

Numerous studies have found cervical cancer stigma to be a significant barrier to uptake of screening services [14–17]. Manifestations of this stigma can range from rejection to exclusion, blame, or devaluation resulting from unfavorable social judgment of the patient [18,19].

However, there remains little published research on the measurement of stigma and its effects on cervical cancer screening uptake, particularly among women in Senegal. Additionally, a better understanding of stigma in urban and rural settings is needed in order to adapt screening strategies and improve screening uptake for Senegalese women. This study aims to develop a comprehensive stigma index and assess the association between cervical cancer-related stigma and prior cervical cancer screening uptake among women in the Dakar and Kédougou regions of Senegal.

### Study framework

This cross-sectional quantitative study was carried out in the Dakar and Kédougou regions of Senegal. The country has a population of around 18 million, unevenly distributed across its 14 administrative regions [20]. The Dakar region accounts for 22% of the national population, with a density of 7,277 inhabitants/km², while Kédougou is the least populated, with a density of just 15 inhabitants/km² [21].

In economic terms, Senegal has significant economic disparities: 53.6% of the rural population lives below the national poverty line, compared with 19.8% in urban areas, and 75.4% of the nation’s poor live in rural areas [22]. The gross enrolment rate (GER), which assesses access to schooling for individuals by cycle, stands at 18.2% for preschool, 81.0% for primary, 50.6% for intermediate and 30.3% for secondary. Across all education levels, the GER is higher in urban areas than in rural areas, regardless of the level of education [21].

Senegal’s healthcare system includes 44 public health care centers. Almost all major health facilities are located in the Dakar region, which comprises 12 health districts and also concentrates the majority of human resources dedicated to health care (38.9% of staff). Kédougou, by comparison, has three health districts and just 1.4% of qualified staff [23]. In the Dakar region the study was conducted in the districts of Keur Massar, Diamniadio, and Yeumbeul. In the Kedougou region all the three districts (Kédougou, Saraya, and Salemata) were included. Each of these health districts has a single health center in the district capital and several health posts in the surrounding rural communities.

Cervical cancer screening is integrated into the national reproductive health program and delivered through a decentralized health system. Screening services are available at the district level, including health centers and district hospitals and are also provided at health posts depending on staff training and equipment availability. The standard screening method recommended by the Ministry of Health is visual inspection with acetic acid (VIA), sometimes combined with visual inspection with Lugol’s iodine (VILI), particularly in primary health centers. Cytology (Pap smear) and HPV testing are available in selected referral hospitals, mainly in urban areas such as Dakar.

### Materials and methods

#### Ethics statement.

This project is part of a patient navigation program [24] and is guided by a research partnership between the Senegal Ministry of Health and Social Action, Cheikh Anta Diop (UCAD) University in Dakar and the University of Illinois Chicago (UIC). The human subjects research has been approved by the UIC Cancer Center Protocol Review Committee (protocol number 2022–0055), the UIC Institutional Review Board (protocol number 2021–1282), and the National Ethics Committee for Health Research at the Senegal Ministry of Health and Social Action (protocol number SN22/133). This National Institutes of Health, National Cancer Institute funded grant (R01CA258683, GRANT13412230) is registered as #NCT5544084 on clinicaltrials.gov.

Prior to the start of the study, written informed consent was obtained from all participants by means of a written consent procedure in a language that the participant could understand.

**Sample and sampling procedure:** A two-stage cluster design was used to sample study participants across the six health districts in the Dakar and Kédougou regions. In the first stage, three reference health centers (at the district level) were identified in each of the two regions. For each center, we randomly selected two additional satellite health posts within geographical proximity. Census districts associated with these health centers and health posts were the unit of sampling for the first stage. In the second stage of sampling, we identified households within the census districts with at least one woman aged 25–69 years eligible for cervical cancer screening, and randomly selected female and male participants from a subset of these households. A total of 720 participants, comprising 360 male–female couples, were uniformly sampled from the catchment areas of the six health districts comprising health centers and posts. Because the present analysis focused on cervical cancer screening uptake, only female participants were included. Of the 360 women enrolled in the parent study, 287 had complete data on prior cervical cancer screening and were retained for analysis. Women with missing information on screening history were excluded. Participants were not compensated for their time in accordance with local research practices.

**Survey data:** Quantitative data were collected using surveys administered by trained research staff in the participant’s preferred language (French, Malinké, or Pulaar). Surveys included socio-demographic characteristics (i.e., age, marital status, and education). Data on potential clinical and behavioral risk factors included oral contraceptive use, age at first pregnancy, and number of live births.

**Primary outcome:** Past participation in cancer screening was the primary outcome of this study and was based on self-reported responses to the question, “Have you ever been screened for cervical cancer?” The response options were “No, I’ve never been screened”, “Yes once”, “Yes more than once” and “Don’t know/ Not sure”. A dichotomous screening variable was subsequently created and women who reported one or more past screenings were categorized as having been screened.

**Stigma index:** We have used a well-established methodological approach to develop the stigma index [13,25,26] and quantified the level of cervical cancer stigma in the study population using a set of 34 survey items, each rated on a five-point Likert scale (strongly disagree to strongly agree; coded 0–4). The survey items (S1 Table) span several domains that have been shown to be strong predictors of stigma, including religious attitudes, shame, avoidance, awkwardness, trust in healthcare systems, and rumors about HPV vaccination and cervical cancer [14,16]. The Receiver Operating Characteristic curve (ROC) presents the sensitivity, specificity, and predictive power of the stigma scores based on a logistic model of cervical cancer screening, adjusted for age, marital status and education (S1 Fig). The calibration plot demonstrates strong agreement between the observed and model-predicted probabilities of cervical cancer screening (S2 Fig). Together, the ROC and calibration plot demonstrate the strong predictive power of the stigma index.

**Statistical analysis:** Descriptive summaries were reported using means and standard deviations for numeric data and frequencies and percentages for categorical variables, both in the overall sample and stratified by the primary outcome (prior cervical cancer screening). Bivariate associations between the outcome and study variables were assessed for significance using t-tests and chi-squared for numeric and categorical predictors, respectively.

We applied a structural equation modeling (SEM) approach to develop an index for measuring stigma based on responses to relevant survey items. Specifically, we estimated values of the index by implementing exploratory factor analysis (EFA) models. Due to the presence of categorical survey items, we used diagonally weighted least squares extraction. An oblique *promax* rotation was used to allow for correlated factors. The appropriate number of factors was determined using a Scree plot and model fit statistics were assessed using standard indices. Scree plots and goodness-of-fit measures (CFI, TLI, RMSEA, AIC) were used to optimize the number of factors and assess overall model performance. To handle missing values, we used full information maximum likelihood (FIML) to fit the EFA models; multiple imputations were also utilized to validate the results of the FIML approach (S3 Fig). We assessed the effect of stigma on cervical cancer screening using multivariate logistic regression. Models were adjusted for potential confounders identified through a combination of *a priori* knowledge and data-driven methods. Age was included in all models based on prior literature and subject-matter knowledge. Additional covariates were evaluated using a backward selection procedure, and marital status was retained based on the results of this procedure and its impact on the coefficient of the stigma index. We assessed model fit using likelihood measures and computed adjusted odds ratios (ORs) and their 95% confidence intervals (95% CIs). P-values were calculated for all inferential methods and the significance of hypotheses were assessed using an alpha = 0.05 threshold. Analyses were conducted in R version 4.5.0 (R Core Team, 2025) and SEM methods were implemented using the *lavaan* package [27].

## Human subjects research approval

### Results

Among the 287 women with available screening data, only 15.7% reported a history of cervical cancer screening, highlighting the low level of screening uptake in this population. The mean age of participants was 36.2 years, and the majority (68.6%) were married and living in monogamous households. Compared to unscreened women, a greater proportion of screened women received some formal education (80% and 59.5%, respectively, p = 0.016) and lived in urban regions (73.3% and 51.2%, respectively, p = 0.011). The proportion of participants with a prior history of screening was significantly higher in urban than in rural areas (73.3% vs. 26.7%, respectively; p = 0.01). This pattern was consistent across the urban districts of Yeumbeul, Keur Massar, and Diamniadio compared with the rural districts of Salemata, Saraya, and Kédougou (p = 0.011). Most notably, women who had not been screened had positive (high) stigma scores (0.35, SD = 1.41) whereas women who had been screened had negative (low) stigma score scores (-0.38, SD = 1.04, p < 0.001) (Table 1). Fig 1 presents the distribution of stigma scores across the six districts with the vertical line representing median stigma. Positive (high) stigma scores were consistently observed across all three rural districts, whereas urban districts tended to show negative (low) stigma scores, albeit with higher median stigma levels. Fig 2 presents the predicted rates of prior cervical cancer screening—adjusted for age, marital status, and education—plotted against stigma scores. The results demonstrate a strong relationship between stigma and screening: as stigma scores increase, predicted screening rates decline. Further, the ROC and calibration analyses results underscore the predictive power of the stigma index in modeling cancer-related health-seeking behavior and attitudes. On the other hand, the close alignments between empirical factors that comprise the index and *a priori* stigma domains emphasize the validity of the index

**Table 1:** Selected characteristics, by screening status (n = 287).

	Overall(N = 287)	Not screened(N = 242)	Screened(N = 45)	P-values
**Stigma index score**	0.24 (1.38)	0.35 (1.41)	-0.38 (1.04)	<0.001
**Age (years)**	36.23 (9.78)	35.80 (9.35)	38.51 (11.68)	0.15
Missing	1 (0.3%)	1 (0.4%)	0 (0%)	
**Marital status**				
Married (Monogamous household)	197 (68.6%)	168 (69.4%)	29 (64.4%)	0.45
Married (Polygamous household)	70 (24.4%)	56 (23.1%)	14 (31.1%)	
Unmarried (Single/Divorced/Widowed)	20 (7.0%)	18 (7.4%)	2 (4.4%)	
**Formal education**				
At least some^a^	180 (62.7%)	144 (59.5%)	36 (80.0%)	0.016
None	106 (36.9%)	97 (40.1%)	9 (20.0%)	
Missing	1 (0.3%)	1 (0.4%)	0 (0%)	
**Oral Contraception Use**				
No	180 (62.7%)	155 (64.0%)	25 (55.6%)	0.36
Yes	107 (37.3%)	87 (36.0%)	20 (44.4%)	
**Age at First Pregnancy**	20.11 (4.99)	20.05 (4.95)	20.38 (5.22)	0.72
Missing	49 (17.1%)	44 (18.2%)	5 (11.1%)	
**Number of live births**				
None	12 (4.2%)	10 (4.1%)	2 (4.4%)	0.56
1-3	129 (44.9%)	105 (43.4%)	24 (53.3%)	
4+	138 (48.1%)	119 (49.2%)	19 (42.2%)	
Missing	8 (2.8%)	8 (3.3%)	0 (0%)	
**Urban/rural location**				
Rural	130 (45.3%)	118 (48.8%)	12 (26.7%)	0.01
Urban	157 (54.7%)	124 (51.2%)	33 (73.3%)	
**Senegal district**				
Yeumbeul	48 (16.7%)	34 (14.0%)	14 (31.1%)	0.011
Keur Massar	54 (18.8%)	43 (17.8%)	11 (24.4%)	
Diamniado	55 (19.2%)	47 (19.4%)	8 (17.8%)	
Salemata	41 (14.3%)	34 (14.0%)	7 (15.6%)	
Saraya	34 (11.8%)	31 (12.8%)	3 (6.7%)	
Kedougou	55 (19.2%)	53 (21.9%)	2 (4.4%)	

^a^Includes primary or secondary school or higher education.

**Fig 1:**
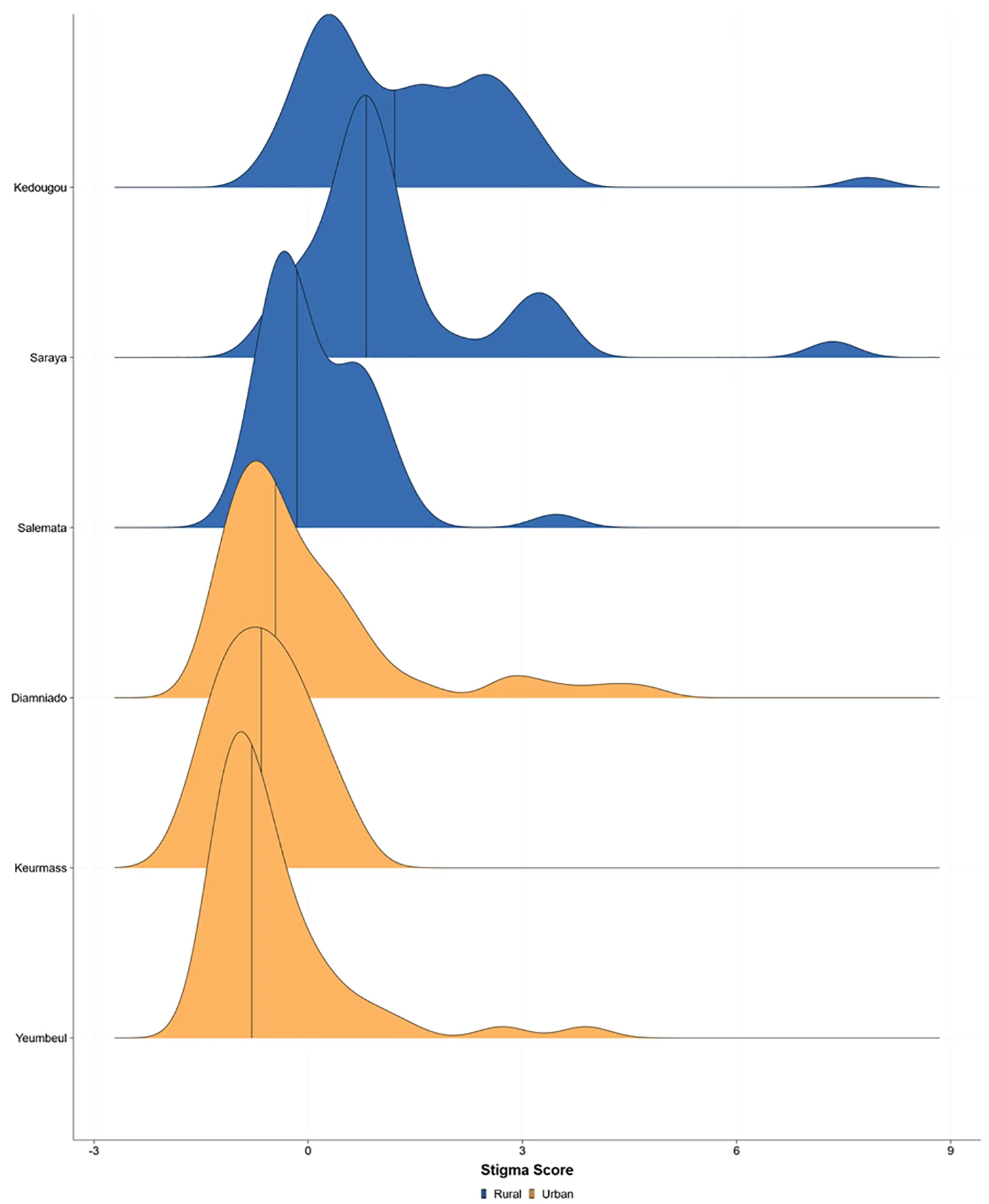
Distribution of stigma scores by urban and rural districts.

**Fig 2:**
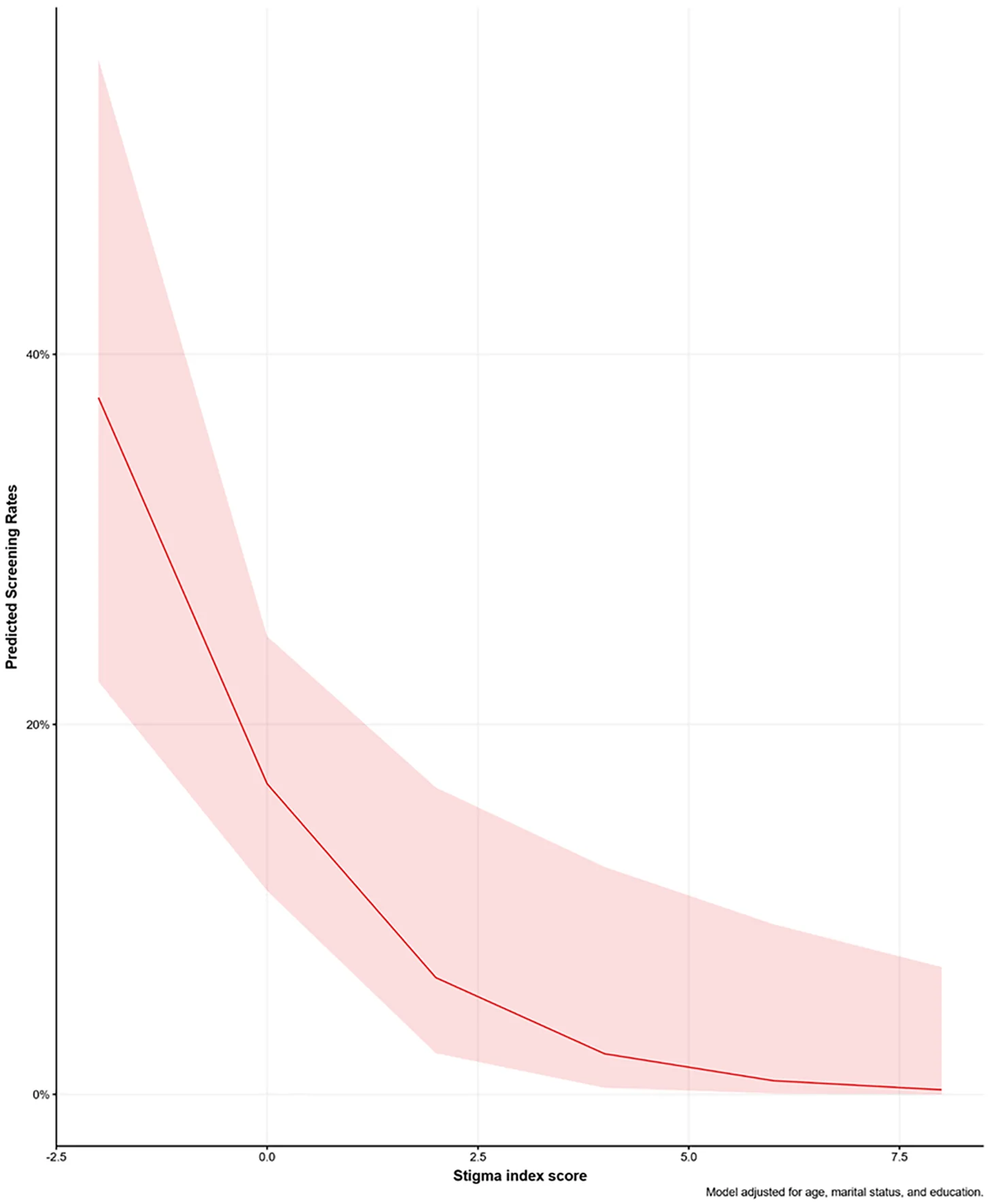
Predicted rates of cervical cancer screening by stigma score.

### Logistic regression results

Four logistic models were fitted to explore the association between stigma and the odds of having been screened in the past for cervical cancer (S4 Fig). Both the crude and age-adjusted models produced similar and statistically significant results, indicating that as stigma increases, the odds of prior cervical cancer screening decrease (model 1 and 2, respectively). The interaction between stigma and urbanicity was not statistically significant (p = 0.49), nor was the covariate for urbanicity (p = 0.47). The final model, derived through manual backward selection, included covariates that were marginally statistically significant (Table 2). After adjusting for age, marital status, and education, higher stigma scores were associated with lower odds of prior screening (OR=0.58, 95% CI 0.38-0.83, p = 0.006).

**Table 2:** Final logistic regression for the relationship between stigma score and cervical cancer screening (N = 285).

Predictors	Odds Ratio (95% CI)	P-value
Stigma index score	0.58	**0.006**
Age(years)	1.03	0.078
Married (Polygamous household)	1.68	0.179
Unmarried (Single/Divorced/Widowed)	0.44	0.312
No Formal Education	0.45	0.059
AIC	237.166	

### Discussion

In this study, cervical cancer screening uptake was low, with only 15.7% of women reporting a previous screening test. We also found that higher levels of cervical cancer-related stigma were significantly associated with lower odds of prior screening. In contrast, educational level and type of residence were not significantly associated with screening uptake. These findings suggest that stigma may represent an important barrier to participation in cervical cancer screening services among women in Senegal.

Cervical cancer screening is an essential secondary prevention strategy in the fight against this disease. The World Health Organization (WHO) recommends that all countries worldwide achieve a cancer screening rate of 70% by 2030 to make progress towards eliminating cervical cancer [28]. In our study, we documented a cervical cancer screening rate of 15.7%, well below the targets recommended by the WHO. This rate is also well below those found in studies carried out in France in 2022 (59.5%) [29] and in Rwanda in 2019 (28.3%) [30]. However, this rate is comparable to that reported in a study conducted in Nigeria, where cervical cancer screening coverage was 15.6% among women of childbearing age [31], but higher than the 6.3% rate observed in a rural study in Senegal [32].

Cancer-related stigma has a negative impact on both patients and the community. It leads to adverse psychosocial and health consequences, a deterioration in patients’ quality of life, delays in diagnosis and in seeking care and treatment, as well as discrimination in the workplace [33]. Our study identified a significant association between cervical cancer stigma and a history of cervical cancer screening. In our study, higher stigma scores were associated with lower odds of prior cervical cancer screening, suggesting that stigma may represent a significant barrier to screening uptake in this population. This finding underscores the need to implement communication strategies to reduce cervical cancer stigma in Senegal, with the goal of improving screening uptake and women’s health outcomes. Our results are in line with those found in a study conducted by Paneru et al. in India in 2021 [15] in which stigma was inversely associated with the practice of cervical cancer screening, and women who perceived cancer as a terminal illness with an inability to return to normal were less likely to undergo screening. Our findings are also similar to those in England [34] and in the Dominican Republic [17] where cervical cancer was taboo and fear and stigma were perceived to negatively affect screening uptake. A study carried out in Kenya in 2022 found that cervical cancer stigma and discriminatory attitudes of community members, including assumptions of promiscuity, infidelity, or HIV status, were perceived to prevent women from accessing screening and treatment opportunities [35]. In Senegal, a study showed the importance of ensuring that women feel empowered to prevent cervical cancer through screening, by ensuring that stigma, attitudes and beliefs are prioritized in awareness-raising efforts to encourage uptake of screening [16].

In our study educational level and type of residence were not significantly associated with prior cervical screening uptake. These results differ from those found in a study carried out in the Kigali district in which educational level was positively associated with screening uptake, and that rural residence was a barrier to cervical cancer screening [30]. Our results also differ from observations made in Nepal, where literate women had a higher rate of cervical cancer screening than others. Another study found that cervical cancer–related stigma was increased by awareness of the sexually transmitted nature of the human papillomavirus (HPV), the primary cause of the disease [36]. In Senegal, a study showed a positive association between French school education and screening uptake (OR = 2.56; CI = [1.72-3.821]) [32].

Our stigma index provides a comprehensive assessment of stigma, incorporating multiple domains known to be strong predictors of health-related stigma. Stigma was measured at baseline to enable examination of its association with prior screening behavior, as well as differences across predefined subgroups (e.g., region, education level, marital status). The index represents a novel tool that can be adapted for future research, including the evaluation of stigma reduction following implementation of our patient navigation intervention. Nevertheless, our study has certain limitations. Not all women initially enrolled in the survey could be included in the analysis due to missing data, and excluding women with missing screening data may have introduced selection bias. Also, the temporality of the relationship between cancer-related stigma and screening uptake was not established due to the cross-sectional nature of the study at baseline. While higher stigma scores were associated with lower odds of screening, it is equally plausible that women who have previously undergone screening may subsequently report lower stigma due to increased knowledge, familiarity with health services, or reassurance following a negative result. This potential reverse causality limits causal interpretation. Also, the low screening uptake observed in our study may reflect structural constraints, including limited availability of services in rural districts, in addition to stigma-related perceptions. Furthermore, our study could be subject to social desirability bias. As an internal and subjective feeling, stigma is complex and difficult to assess comprehensively using explicit measures. Respondents might adjust their answers to conform to social norms or to avoid fully revealing their feelings. Participation in screening in our study was also self-reported, which could also lead to a social desirability bias. In addition to social desirability bias, common-method bias may have influenced the observed association, as both stigma perceptions and screening status were self-reported and measured simultaneously.

### Conclusion

Cervical cancer-related stigma was more prevalent among non-screened women included in our study and was significantly associated with lower odds of screening uptake. A better understanding of this barrier, through qualitative research, could enable screening strategies to be better adapted, specifically targeting women living in rural areas where access to health centers is difficult. Future research should explore whether patient navigation programs for cervical cancer screening could improve access to this service by reducing stigma-related barriers.

## Supporting information

S1 Table Survey items. (DOCX)

S1 Fig Receiver Operating Characteristic (ROC) curve. (TIF)

S2 Fig Calibration plot. (TIF)

S3 Fig Stigma score and comparison of FIML and multiple imputation. (TIF)

S4 Fig Estimated odds ratios (95% CI), models 1–5. (TIF)
