# Supplementary material for: Impact of cervical cancer–related stigma on screening uptake in Dakar and Kedougou, Senegal: A context-specific stigma index
Source: PLOS Glob Public Health. 2026 Oct 8;6(10):e0006187. doi: 10.1371/journal.pgph.0006187 (PMC13648984; doi:10.1371/journal.pgph.0006187)
Supplement: Supplementary file 1 [file pgph.0006187.s001.docx]

S1 Table: Survey Items

| **SECTION** | | | | **SUBSECTION** | | **#** | **QUESTION** | **RESPONSE** |
| --- | --- | --- | --- | --- | --- | --- | --- | --- |
|  | **Administrative** | | | | | |  |  |
|  |  |  |  |  |  | **1** | DATA COLLECTION TIME PERIOD # | 0) T0  2) T2  4) T4  6) T6 |
|  |  |  |  |  |  | **2** | INTERVIEWER | OPEN ENDED RESPONSE |
|  |  |  |  |  |  | **3** | DATE | OPEN ENDED RESPONSE |
|  |  |  |  |  |  | **4** | PARTICIPANT UNIQUE IDENTIFICATION # | OPEN ENDED RESPONSE |
|  |  |  |  |  |  | **5** | GENDER | Female |
|  |  |  |  | **Demographics** | | |  |  |
|  |  |  |  |  |  | **6** | What is your age? | OPEN ENDED RESPONSE |
|  |  |  |  |  |  | **7** | What is the highest level of education that you have completed? | 0) None  1) Primary school  2) Secondary school  3) Higher education  66) PREFER NOT TO ANSWER |
|  |  |  |  |  |  | **8** | What is your marital status? | 1) Single  2) Married (monogamous household)  3) Married (polygamous household)  4) Living as married / cohabitating  5) Divorced or Separated  6) Widowed  66) PREFER NOT TO ANSWER |
|  |  |  |  |  |  | **9** | What languages do you prefer to speak? | SELECT ALL THAT APPLY  1) Malinke  2) Pulaar  3) Basari  4) Bedick  5) Serer  6) Wolof  7) French  88) Other  66) PREFER NOT TO ANSWER |
|  |  |  |  |  |  | **10** | What is your position? | 1) Patient Navigator Leader  2) Nurse  3) Midwife  4) Bajenu Gox  5) Patient / Community Member |
|  |  |  |  |  |  | **11** | Region: | 1) Kedougou  2) Dakar |
|  |  |  |  |  |  | **12** | District: | 1) District Kédougou  2) District Saraya  3) District Salémata  4) District Yeumbeul  5) District Diamniadio  6) District Keur Massar |
|  |  |  |  | **ATTITUDES - RELIGIOUS** | | | **Participants will select among the listed responses or complete the open-ended response.**  **All Likert Scales correspond to the following choices:**  **1) Strongly Disagree**  **2) Somewhat Disagree**  **3) Neutral**  **4) Somewhat Agree**  **5) Strongly Agree**  **66) PREFER NOT TO ANSWER**  **99) DON'T KNOW / NOT SURE** | |
|  |  |  |  |  |  | **37** | Getting a serious disease like cervical cancer is fate, there is nothing I can do to change fate. | 1 \| 2 \| 3 \| 4 \| 5 \| 66 \| 99 |
|  |  |  |  |  |  | **38** | Getting cervical cancer is a curse from god. | 1 \| 2 \| 3 \| 4 \| 5 \| 66 \| 99 |
|  |  |  |  | **ATTITUDES - STIGMA** | | |  |  |
|  |  |  |  |  |  | **39** | Cervical cancer screening is for promiscuous women. | 1 \| 2 \| 3 \| 4 \| 5 \| 66 \| 99 |
|  |  |  |  |  |  | **40** | I would not feel comfortable being around someone with cervical cancer. | 1 \| 2 \| 3 \| 4 \| 5 \| 66 \| 99 |
|  |  |  |  |  |  | **41** | Once you’ve had cervical cancer you are never ‘normal’ again. | 1 \| 2 \| 3 \| 4 \| 5 \| 66 \| 99 |
|  |  |  |  |  |  | **42** | If a person has cervical cancer it’s probably their fault. | 1 \| 2 \| 3 \| 4 \| 5 \| 66 \| 99 |
|  |  |  |  |  |  | **44** | Cervical cancer is more frightening than most other diseases. | 1 \| 2 \| 3 \| 4 \| 5 \| 66 \| 99 |
|  |  |  |  |  |  | **45** | If I had cervical cancer, I would feel ashamed. | 1 \| 2 \| 3 \| 4 \| 5 \| 66 \| 99 |
|  |  |  |  |  |  | **46** | If I had cervical cancer, some people in my family would feel ashamed. | 1 \| 2 \| 3 \| 4 \| 5 \| 66 \| 99 |
|  |  |  |  |  |  | **47** | If someone else in my family had cervical cancer, I would feel ashamed. | 1 \| 2 \| 3 \| 4 \| 5 \| 66 \| 99 |
|  |  |  |  | **ATTITUDES - SOCIAL** | | |  |  |
|  |  |  |  |  |  | **48** | Other women who have gotten the cervical cancer test have reported that it was comfortable. | 1 \| 2 \| 3 \| 4 \| 5 \| 66 \| 99 |
|  |  |  |  | **RUMORS - CERVICAL CANCER** | | |  |  |
|  |  |  |  |  |  | **53** | Cervical cancer screening is a harmful practice. | 1 \| 2 \| 3 \| 4 \| 5 \| 66 \| 99 |
|  |  |  |  |  |  | **54** | I have heard stories about cervical cancer screening that worry me. | 1 \| 2 \| 3 \| 4 \| 5 \| 66 \| 99 |
|  |  |  |  |  |  | **55** | I know people who say negative things about cervical cancer screening. | 1 \| 2 \| 3 \| 4 \| 5 \| 66 \| 99 |
|  |  |  |  |  |  | **56** | What I hear from others about cervical cancer screening makes me doubt what my healthcare provider is telling me. | 1 \| 2 \| 3 \| 4 \| 5 \| 66 \| 99 |
|  |  |  |  | **HEALTHCARE STRUCTURE - TRUST** | | |  |  |
|  |  |  |  |  |  | **80** | At times, when receiving healthcare, I have been treated badly. | 1 \| 2 \| 3 \| 4 \| 5 \| 66 \| 99 |
|  |  |  |  |  |  | **81** | Patients have sometimes been deceived or misled at the healthcare facility. | 1 \| 2 \| 3 \| 4 \| 5 \| 66 \| 99 |
|  |  |  |  |  |  | **82** | Healthcare facilities have sometimes done harmful experiments on patients without their knowledge. | 1 \| 2 \| 3 \| 4 \| 5 \| 66 \| 99 |
|  |  |  |  |  |  | **83** | Healthcare facilities put the patient’s health first. | 1 \| 2 \| 3 \| 4 \| 5 \| 66 \| 99 |
|  |  |  |  |  |  | **84** | Patients should always follow the advice given to them at the healthcare facility. | 1 \| 2 \| 3 \| 4 \| 5 \| 66 \| 99 |
|  |  |  |  |  |  | **85** | They know what they are doing at the healthcare facility. | 1 \| 2 \| 3 \| 4 \| 5 \| 66 \| 99 |
|  |  |  |  |  |  | **86** | Mistakes are common at the healthcare facility. | 1 \| 2 \| 3 \| 4 \| 5 \| 66 \| 99 |
|  |  |  |  |  |  | **95** | I hear a lot of rumors about the cervical cancer test. | 1 \| 2 \| 3 \| 4 \| 5 \| 66 \| 99 |
|  |  |  |  |  |  | **98** | I would be embarrassed to get the cervical cancer test. | 1 \| 2 \| 3 \| 4 \| 5 \| 66 \| 99 |
|  |  |  |  |  |  | **99** | I am scared to get the cervical cancer test. | 1 \| 2 \| 3 \| 4 \| 5 \| 66 \| 99 |
|  |  |  |  |  |  | **100** | I have concerns about the impact of cervical cancer screening on my ability to have sex. | 1 \| 2 \| 3 \| 4 \| 5 \| 66 \| 99 |
|  |  |  |  |  |  | **101** | Getting the cervical cancer test would go against my religion. | 1 \| 2 \| 3 \| 4 \| 5 \| 66 \| 99 |
|  |  |  |  |  |  | **102** | A family member would not allow me to get the cervical cancer test. | 1 \| 2 \| 3 \| 4 \| 5 \| 66 \| 99 |
|  |  |  |  |  |  | **103** | My friends have discouraged me from getting the cervical cancer test. | 1 \| 2 \| 3 \| 4 \| 5 \| 66 \| 99 |
|  |  |  |  | **RUMORS - HPV VACCINATION** | | |  |  |
|  |  |  |  |  |  | **131** | HPV vaccination is a harmful practice. | 1 \| 2 \| 3 \| 4 \| 5 \| 66 \| 99 |
|  |  |  |  |  |  | **132** | HPV vaccination causes infertility. | 1 \| 2 \| 3 \| 4 \| 5 \| 66 \| 99 |
|  |  |  |  |  |  | **133** | I have heard stories about the HPV vaccine that worry me. | 1 \| 2 \| 3 \| 4 \| 5 \| 66 \| 99 |
|  |  |  |  |  |  | **134** | I know people who say negative things about the HPV vaccine. | 1 \| 2 \| 3 \| 4 \| 5 \| 66 \| 99 |
|  |  |  |  |  |  | **135** | What I hear from others about the HPV vaccine makes me doubt what my healthcare provider is telling me. | 1 \| 2 \| 3 \| 4 \| 5 \| 66 \| 99 |
