# Supplementary material for: Impact of cervical cancer–related stigma on screening uptake in Dakar and Kedougou, Senegal: A context-specific stigma index
Source: PLOS Glob Public Health. 2026 Oct 8;6(10):e0006187. doi: 10.1371/journal.pgph.0006187 (PMC13648984; doi:10.1371/journal.pgph.0006187)
Supplement: Supplementary file 2 [file pgph.0006187.s006.docx]

This is an interesting and original article that could provide insights into improving cervical cancer screening.

In terms of format, the article is very long, especially the introduction, which should be reduced by half. All that is needed are statistical data to show the importance of the problem as a public health priority and the second most common cancer among women in Senegal after breast cancer. The article should then pose the problem and the research hypothesis of stigma as a factor hindering screening.

In terms of form, the authors place references in square brackets after the full stop, when it should be the other way around.

In terms of content:

• The subdivision of health districts needs to be reviewed in both Dakar and Kédougou.

• The description of screening provision as an activity at national and district level (standard screening organisation and screening method, cervical smears, number of laboratories, etc.).

• The methodology, where it is sometimes difficult to know whether it is a qualitative or quantitative study.

• In the discussion, clearly highlight the bias, in particular the low screening rate, which is also linked to the limited availability of screening, particularly in rural areas.

The translation of the questionnaires into the national language was not specified and, from an ethical point of view, were the participants compensated (remunerated) for the time allocated to the administration of the questionnaire ?
