# Supplementary material for: Impact of cervical cancer–related stigma on screening uptake in Dakar and Kedougou, Senegal: A context-specific stigma index
Source: PLOS Glob Public Health. 2026 Oct 8;6(10):e0006187. doi: 10.1371/journal.pgph.0006187 (PMC13648984; doi:10.1371/journal.pgph.0006187)
Supplement: Supplementary file 3 [file pgph.0006187.s008.docx]

Adama Faye	Dakar, Wednesday, Mars 4, 2026

Titulary Professor of Public Health

Cheikh Anta Diop University, Dakar, Senegal

Port: 00221 773246336

Email: adamafaye94@gmail.com

**PLOS Global Public Health**

**Object : Submission letter responses to comments**

Dear Mr or Mrs,

I am submitting the responses to the comments made during the evaluation of the article entitled « ***Impact of cervical cancer–related stigma on screening uptake in Dakar and Kedougou, Senegal: A context-specific stigma index* ».**

This letter includes a table in the appendix summarizing the responses to each comment. These responses are also included in the body of the article, in tracked changes mode (revision).

I hope that these responses are satisfactory. Please do not hesitate to contact me if you require any further information. I look forward to a favorable outcome of the article evaluation, please accept, Mr or Mrs, the assurance of my most sincere consideration.

***Pr Adama FAYE***

**Appendix :**

| **REVIEWERS COMMENTS** | **RESPONSE** |
| --- | --- |
| In terms of format, the article is very long, especially the introduction, which should be reduced by half.  All that is needed are statistical data to show the importance of the problem as a public health priority  and the second most common cancer among women in Senegal after breast cancer. The article should  then pose the problem and the research hypothesis of stigma as a factor hindering screening. | We thank the reviewer for this constructive comment. We have substantially revised and shortened the Introduction section to improve focus and clarity. |
| In terms of form, the authors place references in square brackets after the full stop, when it should be  the other way around. | We have revised the manuscript to ensure that all references are placed before punctuation marks in accordance with journal formatting guidelines. |
| The subdivision of health districts needs to be reviewed in both Dakar and Kédougou. | We thank the reviewer for this comment. We have carefully reviewed and clarified the administrative subdivision of health districts in both Dakar and Kedougou regions. The revised manuscript now accurately describes the number of health districts in each region and specifies the districts included in the study. |
| • The description of screening provision as an activity at national and district level (standard screening organisation and screening method, cervical smears, number of laboratories, etc.). | Section added to the manuscript:  Cervical cancer screening is integrated into the national reproductive health program and delivered through a decentralized health system. Screening services are available at the district level, including health centers and district hospitals, but are also provided at health posts depending on staff training and equipment availability. The standard screening method recommended by the Ministry of Health is visual inspection with acetic acid (VIA), sometimes combined with visual inspection with Lugol’s iodine (VILI), particularly in primary health centers. Cytology (Pap smear) and HPV testing are available in selected referral hospitals, mainly in urban areas such as Dakar. |
| • The methodology, where it is sometimes difficult to know whether it is a qualitative or quantitative study. | We have revised (in bold) the first sentence of the “Study framework” as well as the first sentence of the “Survey data” section as follows: This cross-sectional **quantitative** study was carried out in the Dakar and Kedougou regions of Senegal.  **Quantitative** data were collected using **surveys** administered by trained research staff in the preferred language (French, Malinké, or Pulaar) of participants. |
| In the discussion, clearly highlight the bias, in particular the low screening rate, which is also linked to the limited availability of screening, particularly in rural areas. | We have revised the Discussion section to highlight this bias, address potential reverse causality, and note the consequences of structural constraints on screening, as follows: While higher stigma scores were associated with lower odds of screening, it is equally plausible that women who have previously undergone screening may subsequently report lower stigma due to increased knowledge, familiarity with health services, or reassurance following a negative result. This potential reverse causality limits causal interpretation. Also, the low screening uptake observed in our study may reflect structural constraints, including limited availability of services in rural districts, in addition to stigma-related perceptions. |
| The translation of the questionnaires into the national language was not specified and, from an ethical point of view, were the participants compensated (remunerated) for the time allocated to the administration of the questionnaire? | It is stated in the manuscript that “Data were collected using questionnaires administered by trained research staff **in the preferred language (French, Malinké, or Pulaar) of participants**.” The participants were not compensated for the time allocated to the administration of the questionnaire, and we have added the following sentence to clarify this: **Participants were not compensated for their time in accordance with local research practices.** |
| . However, the analytical claims substantially outpace what the data and design can support. The manuscript relies on cross-sectional, self-reported data, yet repeatedly implies causality that is not empirically established.  What the analysis actually demonstrates is an association between the constructed stigma score and self-reported prior screening. Temporality is not established, which is acknowledged in the limitations but the authors need to go further then this. At the very minimum, the strong causal framing throughout the abstract, discussion, and conclusion needs to be removed. This study is more aligned with association or hypothesis generating. The authors should more explicitly discuss reverse causality  I have some concerns about common-method bias. Both stigma and screening status are self-reported, measured at the same time and subject to social desirability bias. It could be that women who are comfortable enough to screen may also be more comfortable expressing low stigma attitudes. That does not mean stigma caused screening behavior. Again, I know social desirability bias is acknowledged is the limitations, but its implications and weight need to either be better addressed and justified throughout the paper. | We thank the reviewer for these important and thoughtful comments. We agree that the cross-sectional design of our study does not allow for causal inference and that the analysis demonstrates an association between the constructed stigma score and self-reported prior screening rather than a causal relationship. In response, we have carefully revised the manuscript to remove causal language throughout the abstract, results, discussion, and conclusion. The findings are now consistently framed as associations.  In addition, we have substantially expanded the discussion of reverse causality, acknowledging that women who have previously undergone screening may subsequently report lower stigma due to increased knowledge, familiarity with health services, or positive interactions with providers.  Furthermore, we have strengthened the limitations section to explicitly address common-method bias. Because both stigma perceptions and screening status were self-reported and measured at the same time, shared method variance and social desirability bias may have influenced the observed association.  We believe these revisions enhance the methodological transparency and interpretive caution of the manuscript |
| 2. The authors use SEM and exploratory factor analysis to build the stigma index, but the reporting is far too thin for replication or evaluation. More information about factor loadings, internal consistency or reliability, measurement invariances across urban and rural settings, etc. is warranted as the stigma index is a key deliverable and novelty of this study. Right now, the stigma index is like a black box that conveniently produces a statistically significant odds ratio.  . The manuscript uses a relatively sophisticated analytical approach. However, the substantive contribution of all these complex methods is limited. The results ultimately rest on a single, directionally expected association between the composite stigma score and self-reported screening uptake. As presented, it is unclear whether the added methodological complexity meaningfully advances understanding beyond what might be obtained using a simpler summed scale or conventional regression approach. Basically, the analysis feel overengineered for what is, substantively, a single modest association.  Either simplify the analysis and report what is only really relevant, or upgrade analysis to report on new findings. | We have used a well-established methodological approach to develop the scale and have provided citations in the Methods section.  We believe that the value of this scale lies in the application of a more comprehensive measure of stigma that includes components known to be associated with health-related stigma. Further, by measuring stigma at baseline, we are not only able to explore its association with prior screening and subgroup (i.e., region, education, marital status) differences observed in the study population but also with subsequent screening uptake at later timepoints. We have added additional content to the Discussion section as follows:  **Our stigma index provides a comprehensive assessment of stigma, incorporating multiple domains known to be strong predictors of health-related stigma. Stigma was measured at baseline to enable examination of its association with prior screening behavior, as well as differences across predefined subgroups (e.g., region, education level, marital status).**  We have also added additional content in the Results section, as follows:  **Figure 1 presents the distribution of stigma scores across the six districts with the vertical line representing median stigma. Positive (high) stigma scores were consistently observed across all three rural districts, whereas urban districts tended to show negative (low) stigma scores, albeit with higher median stigma levels. Figure 2 presents the predicted rates of prior cervical cancer screening—adjusted for age, marital status, and education—plotted against stigma scores. The results demonstrate a strong relationship between stigma and screening: as stigma scores increase, predicted screening rates decline.** |
| 5. Lastly, there are some grammar and spelling errors throughout. Another proofread would be beneficial. | We have carefully proofread the manuscript and corrected grammatical and typographical errors throughout. |
