# Supplementary material for: Impact of cervical cancer–related stigma on screening uptake in Dakar and Kedougou, Senegal: A context-specific stigma index
Source: PLOS Glob Public Health. 2026 Oct 8;6(10):e0006187. doi: 10.1371/journal.pgph.0006187 (PMC13648984; doi:10.1371/journal.pgph.0006187)
Supplement: Supplementary file 4 [file pgph.0006187.s009.docx]

**Subject: Submission of Revised Manuscript and Response to Reviewers**

Dear Editor,

Please find attached the revised version of our manuscript entitled **"Impact of Cervical Cancer–Related Stigma on Screening Uptake in Dakar and Kedougou, Senegal: A Context-Specific Stigma Index"**, together with our detailed responses to the reviewers' comments.

We have carefully considered each comment and revised the manuscript accordingly. A point-by-point response is provided in the attached response table.

We hope that the revisions satisfactorily address the reviewers' concerns and improve the quality of the manuscript. Should you require any additional information or clarification, please do not hesitate to contact us.

Thank you for your time and consideration. We look forward to your favorable evaluation of our revised manuscript.

Yours sincerely,

| **REVIEWERS COMMENTS** | **RESPONSE** |
| --- | --- |
| Title 1. The title is informative and accurately reflects the study objective. 2. Consider whether the development of the stigma index represents a major contribution of the manuscript. If so, the title could be revised to emphasize both the development and application of the index (“Development and Application of a Context-Specific Stigma Index”). | We appreciate this suggestion. However, the primary objective of this manuscript was to examine the association between cervical cancer–related stigma and screening uptake. The development of the stigma index served as a means to operationalize stigma within the study context. We therefore chose to retain the current title, which we believe better reflects the focus of the manuscript. We have, however, strengthened the description of the index development process throughout the Methods section. |
| Abstract  1. Consider replacing the phrase “early detection” with “screening and treatment of precancerous lesions” for greater precision and consistency with current cervical cancer prevention terminology. | We thank the reviewer for this helpful suggestion. We agree that the phrase "screening and treatment of precancerous lesions" is more precise and aligns better with current cervical cancer prevention terminology. Accordingly, we have revised the abstract and other relevant sections of the manuscript by replacing "early detection" with "screening and treatment of precancerous lesions. |
| Clarify participant flow from the original sample (720) to the final analytical sample (287). The reasons for exclusion should be clearly described, and the potential for selection bias should be discussed. | We thank the reviewer for this comment and agree that the derivation of the analytical sample required clarification. The parent study enrolled 720 participants, comprising 360 male–female couples (360 women and 360 men). Because the objective of the present analysis was to examine the association between cervical cancer-related stigma and cervical cancer screening uptake, only female participants were eligible for inclusion in this analysis. Among the 360 women enrolled, 287 had complete data on prior cervical cancer screening and were therefore included in the analytical sample. We have revised the Methods section to clarify the participant selection process. We have also acknowledged the potential for selection bias in the Limitations section, as women with missing screening data were excluded from the analysis. |
| The study employed a two-stage cluster sampling design. Please clarify whether clustering was accounted for during statistical analyses (survey-weighted analyses, cluster-robust standard errors, or multilevel modeling). | To clarify, two-stage cluster sampling was utilized to sample from the catchment areas of the health posts participating in the parent study. We anticipate and account for clustering effects in the primary hypotheses of the parent study (intervention efficacy) but not for this analysis since it is based on baseline data only.  Survey weighted analysis was not used as the study is not intended to reflect the general Senegalese female population; that is, this was convenience sampling and not representative sampling. |
| Provide a more detailed description of the exploratory factor analysis (EFA) procedures used to construct the stigma index, including factor extraction methods, rotation methods, criteria for factor retention, factor loadings, and model fit statistics. | The EFA model was implemented using the lavaan package in R. We have added the following details: Due to the presence of categorical survey items, we used diagonally weighted least squares extraction. An oblique *promax* rotation was used to allow for correlated factors. The appropriate number of factors was determined using a Scree plot and model fit statistics were assessed using standard indices. |
| Reconsider the interpretation of ROC and calibration analyses as evidence of index validity. These analyses demonstrate predictive performance but do not, by themselves, establish construct validity of the stigma index. | We agree with this assessment, and we have noted this distinction in the manuscript as follows: The ROC and calibration analyses results underscore the predictive power of the stigma index in modeling cancer-related health-seeking behavior and attitudes. On the other hand, the close alignments between empirical factors that comprise the index and *a priori* stigma domains emphasize the validity of the index. |
| The confounder selection strategy requires further justification. Reliance on backward selection alone may not be sufficient for identifying confounders. Please clarify whether variables were selected a priori based on existing literature and subject-matter knowledge. | We have revised the Methods section to clarify that potential confounders were identified using a combination of prior knowledge based on the literature and data-driven methods. Specifically, we clarify that age was included in all models based on prior literature and that covariates were evaluated using backward selection, with marital status retained based on the selection procedure and its impact on the coefficient of the stigma index. |
| The prevalence of cervical cancer screening (15.7%) should be highlighted more prominently in the Results section. | We agree that the prevalence of cervical cancer screening is an important finding. We have revised the Results section to more prominently highlight that only 15.7% of women reported having ever undergone cervical cancer screening. |
| Please provide a participant flow diagram | This analysis used baseline data from the parent study, and we believe that a flow diagram is not applicable in his case. The sample is now more completely described in the *Materials and Methods: Sample and sampling procedure subsection* |
| Given the relatively small number of screening events, model stability may be a concern. Please comment on the robustness of the regression models and the events-per-variable ratio. | We appreciate the opportunity to clarify: The events-per-variable (EPV) ratio suggests that a logistic model with about four predictors is stable, based on guidelines in the literature. While our model variables occupy five degrees-of-freedom (due to the multi-category marital status variable), the primary association between stigma and screening remained consistent in other more parsimonious models, including a crude assessment of the bivariate association. The inclusion of marital status and age was necessary to remain consistent with related literature and based on their conceptual roles as potential confounders. |
| The interaction between stigma and urbanicity is reported as non-significant. Please provide the corresponding interaction coefficient and 95% confidence interval. | To clarify, we assessed relevant interactions during our variable selection process, including the interaction between stigma and urbanicity. None of the interaction terms were statistically significant so were not included in subsequent models. As a result, the corresponding interaction coefficients and 95% confidence intervals were not presented. |
| Consider presenting the screening proportions for each district rather than only reporting differences between urban and rural districts. This would provide a clearer picture of geographical variation in screening uptake. | We have revised our narrative to describe the geographical variation presented in Table 1 as follows:  The proportion of participants with a prior history of screening was significantly higher in urban than in rural areas (73.3% vs. 26.7%, respectively; p=0.01). This pattern was consistent across the urban districts of Yeumbeul, Keur Massar, and Diamniadio compared with the rural districts of Salémata, Saraya, and Kédougou (p=0.011). |
| Please discuss the potential impact of the relatively small number of screening events on model stability and statistical power. | Please see the previous comment |
| Discussion 1. The Discussion section would benefit from beginning with a concise summary of the main findings before comparing them with previous literature. | We thank the reviewer for this helpful suggestion. We have revised the opening of the Discussion section to include a concise summary of the study's main findings before comparing them with previous literature. Specifically, we now highlight the low prevalence of cervical cancer screening uptake (15.7%), the inverse association between cervical cancer-related stigma and screening participation, and the lack of significant associations between screening uptake and educational level or place of residence. |
| The statement in lines 314–315 is not supported by the study design. The study did not assess cervical cancer prevalence; rather, it evaluated cervical cancer screening uptake and stigma. The conclusion should therefore focus on the observed association between stigma and screening behavior. | We have revised the relevant section of the manuscript to ensure that our conclusions are limited to the observed association between stigma and prior screening behavior and do not imply effects on cervical cancer occurrence or prevalence. |
| Recommendations regarding patient navigation programs should be framed as future research directions rather than evidence-based conclusions, as the effectiveness of such interventions was not evaluated in the present study. | We appreciate this comment and agree that our study did not evaluate the effectiveness of patient navigation interventions. We have therefore revised the and Conclusion sections to present patient navigation programs as a potential avenue for future research and intervention development rather than as evidence-based recommendations supported by the current study. |
