# Supplementary material for: Impact of cervical cancer–related stigma on screening uptake in Dakar and Kedougou, Senegal: A context-specific stigma index
Source: PLOS Glob Public Health. 2026 Oct 8;6(10):e0006187. doi: 10.1371/journal.pgph.0006187 (PMC13648984; doi:10.1371/journal.pgph.0006187)
Supplement: Peer review history

# Peer review: Decision Letter 0

**DOI:** 10.1371/journal.pgph.0006187.r001

**Authors:** Peter James

18 Jan 2026

PGPH-D-25-03102

Impact of cervical cancer–related stigma on screening uptake in Dakar and Kedougou, Senegal: A context-specific stigma index

PLOS Global Public Health

Dear Dr. Adama Faye,

Thank you for submitting your manuscript to PLOS Global Public Health. After careful consideration, we feel that it has merit but does not fully meet PLOS Global Public Health’s publication criteria as it currently stands. Therefore, we invite you to submit a revised version of the manuscript that addresses the points raised during the review process.

Please submit your revised manuscript by **6th February 2026** . If you will need more time than this to complete your revisions, please reply to this message or contact the journal office at globalpubhealth@plos.org. When you're ready to submit your revision, log on to https://www.editorialmanager.com/pgph/ and select the 'Submissions Needing Revision' folder to locate your manuscript file.

Please include the following items when submitting your revised manuscript:

- A letter that responds to each point raised by the editor and reviewer(s). You should upload this letter as a separate file labeled 'Response to Reviewers'.
- A marked-up copy of your manuscript that highlights changes made to the original version. You should upload this as a separate file labeled 'Revised Manuscript with Track Changes'.
- An unmarked version of your revised paper without tracked changes. You should upload this as a separate file labeled 'Manuscript'.

Guidelines for resubmitting your figure files are available below the reviewer comments at the end of this letter.

We look forward to receiving your revised manuscript.

Kind regards,

Peter Bai James, PhD

Academic Editor

PLOS Global Public Health

Journal Requirements:

1. Please provide a detailed online Financial Disclosure statement. This is published with the article. It must therefore be completed in full sentences and contain the exact wording you wish to be published.

a) Please clarify all sources of financial support for your study. List the grants, grant numbers, and organizations that funded your study, including funding received from your institution. Please note that suppliers of material support, including research materials, should be recognized in the Acknowledgements section rather than in the Financial Disclosure.

b) State the initials, alongside each funding source, of each author to receive each grant. For example: “This work was supported by the National Institutes of Health (####### to AM; ###### to CJ) and the National Science Foundation (###### to AM).”

c) State what role the funders took in the study. If the funders had no role in your study, please state: “The funders had no role in study design, data collection and analysis, decision to publish, or preparation of the manuscript.”

For more information, please go to our submission guidelines:

https://journals.plos.org/globalpublichealth/s/submission-guidelines#loc-financial-disclosure-statement

2. Please ensure that the funders and grant numbers match between the Financial Disclosure field and the Funding Information tab in your submission form. Note that the funders must be provided in the same order in both places as well.

3. Please update your online Competing Interests statement. If you have no competing interests to declare, please state: “The authors have declared that no competing interests exist.”

4. In the online submission form, you indicated that "Mixed methods data will be available upon request from the corresponding author.".

All PLOS journals now require all data underlying the findings described in their manuscript to be freely available to other researchers, either

a) In a public repository,

b) Within the manuscript itself, or

d) Uploaded as supplementary information.

This policy applies to all data except where public deposition would breach compliance with the protocol approved by your research ethics board. If your data cannot be made publicly available for ethical or legal reasons (e.g., public availability would compromise patient privacy), please explain your reasons by return email and your exemption request will be escalated to the editor for approval. Your exemption request will be handled independently and will not hold up the peer review process, but will need to be resolved should your manuscript be accepted for publication. One of the Editorial team will then be in touch if there are any issues.

For further assistance, you may go to: http://journals.plos.org/globalpublichealth/s/data-availability

5. Please provide separate main figure files in .tif or .eps format only and remove any figures embedded in your manuscript file. Please also ensure that all files are under our size limit of 10MB. Please leave the figure captions in the manuscript.

For more information about how to convert your figure files please see our guidelines: https://journals.plos.org/globalpublichealth/s/figures

6. We notice that your supplementary figures are uploaded with the file type 'Figure'. Please amend the file type to 'Supporting Information'. Please ensure that each Supporting Information file has a legend listed in the manuscript before or after the references list.

7. Please ensure that the Author List in your manuscript file matches the Author List in the online submission form, including initials and order.

8. We notice that your supplementary figures and tables are included in the manuscript file. Please remove them and upload them with the file type 'Supporting Information'. Please ensure that each Supporting Information file has a legend listed in the manuscript before or after the references list.

9. If the reviewer comments include a recommendation to cite specific previously published works, please review and evaluate these publications to determine whether they are relevant and should be cited. There is no requirement to cite these works unless the editor has indicated otherwise.

Additional Editor Comments (if provided):

[Note: HTML markup is below. Please do not edit.]

Reviewers' comments:

Reviewer's Responses to Questions

**Comments to the Author**

1. Does this manuscript meet PLOS Global Public Health’s publication criteria?

Reviewer #1: Partly

Reviewer #2: Partly

**********

2. Has the statistical analysis been performed appropriately and rigorously?

Reviewer #1: I don't know

Reviewer #2: Yes

**********

3. Have the authors made all data underlying the findings in their manuscript fully available (please refer to the Data Availability Statement at the start of the manuscript PDF file)?

The PLOS Data policy

Reviewer #1: Yes

Reviewer #2: No

**********

4. Is the manuscript presented in an intelligible fashion and written in standard English?

Reviewer #1: Yes

Reviewer #2: Yes

**********

Reviewer #1: This is an interesting and original article that could provide insights into improving cervical cancer screening.

In terms of format, the article is very long, especially the introduction, which should be reduced by half. All that is needed are statistical data to show the importance of the problem as a public health priority and the second most common cancer among women in Senegal after breast cancer. The article should then pose the problem and the research hypothesis of stigma as a factor hindering screening.

In terms of form, the authors place references in square brackets after the full stop, when it should be the other way around.

In terms of content:

• The subdivision of health districts needs to be reviewed in both Dakar and Kédougou.

• The description of screening provision as an activity at national and district level (standard screening organisation and screening method, cervical smears, number of laboratories, etc.).

• The methodology, where it is sometimes difficult to know whether it is a qualitative or quantitative study.

• In the discussion, clearly highlight the bias, in particular the low screening rate, which is also linked to the limited availability of screening, particularly in rural areas.

The translation of the questionnaires into the national language was not specified and, from an ethical point of view, were the participants compensated (remunerated) for the time allocated to the administration of the questionnaire ?

Reviewer #2: My recommendation is that this paper needs major revisions, to its analysis, writing/grammar, and language around what it is attempting to do. That said, this manuscript addresses an important and relevant question and makes a potentially valuable contribution via the stigma index. In my opinion, the background and justification for this study is its strength. The analysis, the reporting of findings and discussion need strengthening.

1. However, the analytical claims substantially outpace what the data and design can support. The manuscript relies on cross-sectional, self-reported data, yet repeatedly implies causality that is not empirically established.

What the analysis actually demonstrates is an association between the constructed stigma score and self-reported prior screening. Temporality is not established, which is acknowledged in the limitations but the authors need to go further then this. At the very minimum, the strong causal framing throughout the abstract, discussion, and conclusion needs to be removed. This study is more aligned with association or hypothesis generating. The authors should more explicitly discuss reverse causality

2. The authors use SEM and exploratory factor analysis to build the stigma index, but the reporting is far too thin for replication or evaluation. More information about factor loadings, internal consistency or reliability, measurement invariances across urban and rural settings, etc. is warranted as the stigma index is a key deliverable and novelty of this study. Right now, the stigma index is like a black box that conveniently produces a statistically significant odds ratio.

3. I have some concerns about common-method bias. Both stigma and screening status are self-reported, measured at the same time and subject to social desirability bias. It could be that women who are comfortable enough to screen may also be more comfortable expressing low stigma attitudes. That does not mean stigma caused screening behavior. Again, I know social desirability bias is acknowledged is the limitations, but its implications and weight need to either be better addressed and justified throughout the paper.

4. The manuscript uses a relatively sophisticated analytical approach. However, the substantive contribution of all these complex methods is limited. The results ultimately rest on a single, directionally expected association between the composite stigma score and self-reported screening uptake. As presented, it is unclear whether the added methodological complexity meaningfully advances understanding beyond what might be obtained using a simpler summed scale or conventional regression approach. Basically, the analysis feel overengineered for what is, substantively, a single modest association.

Either simplify the analysis and report what is only really relevant, or upgrade analysis to report on new findings.

5. Lastly, there are some grammar and spelling errors throughout. Another proofread would be beneficial.

**********

what does this mean?). If published, this will include your full peer review and any attached files.

**Do you want your identity to be public for this peer review?**  If you choose “no”, your identity will remain anonymous but your review may still be made public.

For information about this choice, including consent withdrawal, please see our Privacy Policy

Reviewer #1: No

Reviewer #2: No

**********

[NOTE: If reviewer comments were submitted as an attachment file, they will be attached to this email and accessible via the submission site. Please log into your account, locate the manuscript record, and check for the action link "View Attachments". If this link does not appear, there are no attachment files.]

Attachment Submitted filename: review plos One .docx

# Author comment: Author response to Decision Letter 1

**DOI:** 10.1371/journal.pgph.0006187.r002

1 Apr 2026

Attachment Submitted filename: renamed_41e0b.docx

# Peer review: Decision Letter 1

**DOI:** 10.1371/journal.pgph.0006187.r003

**Authors:** Jen Edwards

15 Jun 2026

PGPH-D-25-03102R1

Impact of cervical cancer–related stigma on screening uptake in Dakar and Kedougou, Senegal: A context-specific stigma index

PLOS Global Public Health

Dear Dr. Faye,

Thank you for submitting your manuscript to PLOS Global Public Health. After careful consideration, we feel that it has merit but does not fully meet PLOS Global Public Health’s publication criteria as it currently stands. Therefore, we invite you to submit a revised version of the manuscript that addresses the points raised during the review process.

The manuscript has been evaluated by one reviewer and their comments are available below. The reviewer has raised a number of concerns that need attention. They request clarification and further detail on the statistical analysis and regression models. They also raise a number of concerns over the framing of the conclusions with regards to the study design. Could you please revise the manuscript to carefully address the concerns raised?

Please submit your revised manuscript by Aug 11 2026 11:59PM. If you will need more time than this to complete your revisions, please reply to this message or contact the journal office at globalpubhealth@plos.org. When you're ready to submit your revision, log on to https://www.editorialmanager.com/pgph/ and select the 'Submissions Needing Revision' folder to locate your manuscript file.

Please include the following items when submitting your revised manuscript:

- A letter that responds to each point raised by the editor and reviewer(s). You should upload this letter as a separate file labeled 'Response to Reviewers'.
- A marked-up copy of your manuscript that highlights changes made to the original version. You should upload this as a separate file labeled 'Revised Manuscript with Track Changes'.
- An unmarked version of your revised paper without tracked changes. You should upload this as a separate file labeled 'Manuscript'.

Guidelines for resubmitting your figure files are available below the reviewer comments at the end of this letter.

As the corresponding author, your ORCID iD is verified in the submission system and will appear in the published article. PLOS supports the use of ORCID, and we encourage all coauthors to register for an ORCID iD and use it as well. Please encourage your coauthors to verify their ORCID iD within the submission system before final acceptance, as unverified ORCID iDs will not appear in the published article. *Only*  the individual author can complete the verification step; PLOS staff *cannot*  verify ORCID iDs on behalf of authors.

We look forward to receiving your revised manuscript.

Kind regards,

Jen Edwards

Staff Editor

PLOS Global Public Health

Journal Requirements:

If the reviewer comments include a recommendation to cite specific previously published works, please review and evaluate these publications to determine whether they are relevant and should be cited. There is no requirement to cite these works unless the editor has indicated otherwise.

Additional Editor Comments (if provided):

[Note: HTML markup is below. Please do not edit.]

Reviewers' comments:

Reviewer's Responses to Questions

**Comments to the Author**

Reviewer #3: All comments have been addressed

**********

publication criteria?

Reviewer #3: Partly

**********

3. Has the statistical analysis been performed appropriately and rigorously?

Reviewer #3: Yes

**********

4. Have the authors made all data underlying the findings in their manuscript fully available (please refer to the Data Availability Statement at the start of the manuscript PDF file)?

The PLOS Data policy

Reviewer #3: Yes

**********

5. Is the manuscript presented in an intelligible fashion and written in standard English?

Reviewer #3: Yes

**********

Reviewer #3: Manuscript Title: Impact of Cervical Cancer–Related Stigma on Screening Uptake in Dakar and Kedougou, Senegal: A Context-Specific Stigma Index

This manuscript addresses an important public health issue and provides valuable insights into the relationship between cervical cancer–related stigma and screening uptake in Senegal. The development of a context-specific stigma index is a potentially important contribution. However, clarification of the analytical sample, additional methodological details regarding index construction, and more cautious interpretation of findings are needed to strengthen the manuscript.

Title

1. The title is informative and accurately reflects the study objective.

2. Consider whether the development of the stigma index represents a major contribution of the manuscript. If so, the title could be revised to emphasize both the development and application of the index (“Development and Application of a Context-Specific Stigma Index”).

Abstract

1. Consider replacing the phrase “early detection” with “screening and treatment of precancerous lesions” for greater precision and consistency with current cervical cancer prevention terminology.

Materials and Methods

1. Clarify participant flow from the original sample ( 720) to the final analytical sample (287). The reasons for exclusion should be clearly described, and the potential for selection bias should be discussed.

2. The study employed a two-stage cluster sampling design. Please clarify whether clustering was accounted for during statistical analyses (survey-weighted analyses, cluster-robust standard errors, or multilevel modeling).

3. Provide a more detailed description of the exploratory factor analysis (EFA) procedures used to construct the stigma index, including factor extraction methods, rotation methods, criteria for factor retention, factor loadings, and model fit statistics.

4. Reconsider the interpretation of ROC and calibration analyses as evidence of index validity. These analyses demonstrate predictive performance but do not, by themselves, establish construct validity of the stigma index.

5. The confounder selection strategy requires further justification. Reliance on backward selection alone may not be sufficient for identifying confounders. Please clarify whether variables were selected a priori based on existing literature and subject-matter knowledge.

Results

1. The prevalence of cervical cancer screening (15.7%) should be highlighted more prominently in the Results section.

2. Please provide a participant flow diagram and clarify the reasons for exclusion from the final analytical sample.

3. Given the relatively small number of screening events, model stability may be a concern. Please comment on the robustness of the regression models and the events-per-variable ratio.

4. Lines 265–266: The interaction between stigma and urbanicity is reported as non-significant. Please provide the corresponding interaction coefficient and 95% confidence interval.

5. Lines 253–255: Consider presenting the screening proportions for each district rather than only reporting differences between urban and rural districts. This would provide a clearer picture of geographical variation in screening uptake.

6. Line 250: Please discuss the potential impact of the relatively small number of screening events on model stability and statistical power.

Discussion

1. The Discussion section would benefit from beginning with a concise summary of the main findings before comparing them with previous literature.

2. The statement in lines 314–315 is not supported by the study design. The study did not assess cervical cancer prevalence; rather, it evaluated cervical cancer screening uptake and stigma. The conclusion should therefore focus on the observed association between stigma and screening behavior.

3. Recommendations regarding patient navigation programs should be framed as future research directions rather than evidence-based conclusions, as the effectiveness of such interventions was not evaluated in the present study.

**********

what does this mean?). If published, this will include your full peer review and any attached files.

**Do you want your identity to be public for this peer review?**  If you choose “no”, your identity will remain anonymous but your review may still be made public.

For information about this choice, including consent withdrawal, please see our Privacy Policy

Reviewer #3: **Yes:**  Raikhan Bolatbekova

**********

[NOTE: If reviewer comments were submitted as an attachment file, they will be attached to this email and accessible via the submission site. Please log into your account, locate the manuscript record, and check for the action link "View Attachments". If this link does not appear, there are no attachment files.]

# Author comment: Author response to Decision Letter 2

**DOI:** 10.1371/journal.pgph.0006187.r004

10 Aug 2026

Attachment Submitted filename: Response to Reviewers.docx

# Peer review: Decision Letter 2

**DOI:** 10.1371/journal.pgph.0006187.r005

**Authors:** Julia Robinson

30 Aug 2026

Impact of cervical cancer–related stigma on screening uptake in Dakar and Kedougou, Senegal: A context-specific stigma index

PGPH-D-25-03102R2

Dear Pr Faye,

We are pleased to inform you that your manuscript 'Impact of cervical cancer–related stigma on screening uptake in Dakar and Kedougou, Senegal: A context-specific stigma index' has been provisionally accepted for publication in PLOS Global Public Health.

Before your manuscript can be formally accepted you will need to complete some formatting changes, which you will receive in a follow up email. A member of our team will be in touch with a set of requests.

Please note that your manuscript will not be scheduled for publication until you have made the required changes, so a swift response is appreciated.

IMPORTANT: The editorial review process is now complete. PLOS will only permit corrections to spelling, formatting or significant scientific errors from this point onwards. Requests for major changes, or any which affect the scientific understanding of your work, will cause delays to the publication date of your manuscript.

If your institution or institutions have a press office, please notify them about your upcoming paper to help maximize its impact. If they'll be preparing press materials, please inform our press team as soon as possible -- no later than 48 hours after receiving the formal acceptance. Your manuscript will remain under strict press embargo until 2 pm Eastern Time on the date of publication. For more information, please contact globalpubhealth@plos.org.

Thank you again for supporting Open Access publishing; we are looking forward to publishing your work in PLOS Global Public Health.

Best regards,

Julia Robinson

Executive Editor

PLOS Global Public Health

***********************************************************

Reviewer Comments (if any, and for reference):

Reviewer's Responses to Questions

**Comments to the Author**

Reviewer #3: All comments have been addressed

**********

publication criteria?

Reviewer #3: Yes

**********

3. Has the statistical analysis been performed appropriately and rigorously?

Reviewer #3: Yes

**********

4. Have the authors made all data underlying the findings in their manuscript fully available (please refer to the Data Availability Statement at the start of the manuscript PDF file)?

The PLOS Data policy

Reviewer #3: Yes

**********

5. Is the manuscript presented in an intelligible fashion and written in standard English?

Reviewer #3: Yes

**********

Reviewer #3: Manuscript Title: Impact of cervical cancer–related stigma on screening uptake in Dakar and Kedougou, Senegal: A context-specific stigma index Manuscript Number: Manuscript Title: Impact of cervical cancer–related stigma on screening uptake in Dakar and Kedougou, Senegal: A context-specific stigma index Manuscript Number: PGPH-D-25-03102R1

The authors have satisfactorily and thoroughly addressed all comments and concerns raised during the initial review round. The revised version is substantially improved in terms of methodological clarity, statistical transparency, and cautious interpretation of cross-sectional findings.

Specific Observations:

1. Materials and Methods: The detailed description of the participant flow (clarifying why only the female cohort of n=287 from the parent couple study of n=720 was analyzed) provides the necessary transparency. The methodological expansion of the exploratory factor analysis thoroughly resolves earlier statistical questions.

2. Reframing of Index Validity: The distinction between predictive capability (ROC and calibration plots) and construct validity has been appropriately addressed.

3. Results and Discussion: The prominent placement of the 15.7% screening uptake rate, the structured start of the Discussion, and the cautious reframing of patient navigation programs as future research directions significantly strengthen the academic rigor of the paper.

I have no further substantive comments and congratulate the authors on this valuable contribution to understanding cervical cancer screening barriers and stigma in Senegal.

**********

what does this mean?). If published, this will include your full peer review and any attached files.

**Do you want your identity to be public for this peer review?**  If you choose “no”, your identity will remain anonymous but your review may still be made public.

For information about this choice, including consent withdrawal, please see our Privacy Policy

Reviewer #3: **Yes:**  Raikhan Bolatbekova

**********
